# A Nomogram Based on Molecular Biomarkers and Radiomics to Predict Lymph Node Metastasis in Breast Cancer

**DOI:** 10.3389/fonc.2022.790076

**Published:** 2022-03-15

**Authors:** Xiaoming Qiu, Yufei Fu, Yu Ye, Zhen Wang, Changjian Cao

**Affiliations:** Department of Radiology, Huangshi Central Hospital, Edong Healthcare Group, Affiliated Hospital of Hubei Polytechnic University, Huangshi, China

**Keywords:** lymph node metastasis, breast cancer, molecular biomarkers, radiomics, diagnostics

## Abstract

**Background:**

The aim of this study was to explore the feasibility and efficacy of a non-invasive quantitative imaging evaluation model to assess the lymphatic metastasis of breast cancer based on a radiomics signature constructed using conventional T1-weighted image (T1WI) enhanced MRI and molecular biomarkers.

**Methods:**

Patients with breast cancer diagnosed *via* lymph biopsies between June 2015 and June 2019 were selected for the study. All patients underwent T1WI contrast-enhancement before treatment; lymph biopsy after surgery; and simultaneous Ki-67, COX-2, PR, Her2 and proliferating cell nuclear antigen detection. All images were imported into ITK-SNAP for whole tumor delineation, and AK software was used for radiomics feature extraction. Next, the radiomics signature Rad-score was constructed after reduction of specific radiomic features. A multiple regression logistic model was built by combining the Rad-score and molecular biomarkers based on the minimum AIC.

**Results:**

In all, 100 patients were enrolled in this study, including 45 with non-lymph node (LN) metastasis and 55 with LN metastasis. A total of 1,051 texture feature parameters were extracted, and LASSO was used to reduce the dimensionality of the radiomics features. The log(λ) was set to 0.002786, and 19 parameters were retained for the construction of the radiomics tag Rad-score. ROC was used to evaluate the diagnostic efficiency of Rad-score: the area under the ROC curve (AUC) of the Rad-score for identifying non-lymphatic and lymphatic metastases was 0.891 in the training cohort and 0.744 in the validation cohort. With the incorporation of tumor molecular markers, the AUCs of the training cohort and validation cohort of the nomogram were 0.936 and 0.793, respectively, which were notably higher than the AUCs of the clinical parameters in the training and validation cohorts (0.719 and 0.588, respectively).

**Conclusion:**

The combined model constructed using the Rad-score and molecular biomarkers can be used as an effective non-invasive method to assess LN metastasis of breast cancer. Furthermore, it can be used to quantitatively evaluate the risk of breast cancer LN metastasis before surgery.

## Introduction

Breast cancer is the most common malignant tumor in women globally, with lymphatic metastasis being the main cause of death (1). According American Society of Clinical Oncology Clinical Practice Guideline Update, Axillary lymph node dissection (ALND) is not recommended for patients with breast cancer without nodal metastases and one or two sentinel lymph node metastases (2).The five-year survival rate of patients with axillary lymphatic metastasis is significantly lower than that of patients without lymphatic metastasis (3). Patients with lymphatic metastasis require radiotherapy and chemotherapy in addition to surgery. Pre-treatment method indicating the absence of lymph node metastasis could provide the earlier stage and reduce the mortality for better treatment. Evern through patients under ALND would significant reduce the mortality rate but also improve the morbidities associated with ALND, such as seroma formation, impairment of shoulder movement, neuropathy and arm lymphedema (4). Sentinel lymph node biopsy (SLNB) was used to staging the axilla before treatment. SLN is the first lymphatic drainage lymph node in tumor which lead lymphatic spread (5). But several previous studies have showed that SLNB is the standard method to predict lymph node metastasis but the invasive procedure provide the high false negative rate and other complication (6). For clinical purposes, using effective markers for the individual conditions of different patients with lymph node (LN) metastasis can improve the prognosis of patients by actively adjusting the clinical treatment plan (7). In the previous study, the patients with breast cancer widely accepted complete ALND (cALND) for positive SLN that patients with negative SLN should avoid ALND (8). Several studies have found some biomarkers to predict SLN (9), such as tumor size (10), nucleic acid amplification (CK19) (10), ER status (10) and PR status (11).

Currently, pathological biopsy is the gold standard for identifying LN metastasis in patients with breast cancer. Furthermore, sentinel LN biopsy is a standard clinical procedure for pathological biopsy of patients with breast cancer. However, such methods are invasive, are not readily permitted by patients, and fail to provide comprehensive information regarding metastasis (12, 13).

Currently, the non-invasive method for evaluating breast cancer lymphatic metastasis is mainly based on imaging evaluation. Magnetic resonance imaging (MRI) has high-resolution characteristics, especially in soft tissue contrast, and can accurately display LNs. Radiomics is used to build a mathematical model based on the image data from confirmed cases for high-throughput texture feature data mining, which could then be added to the clinical cases to improve model verification, so as to construct a non-invasive evaluation method for clinical research purposes (14).

In contrast, molecular tumor markers play an important auxiliary role in the clinical diagnosis of tumors. Among them, Ki-67 can react with proliferating nuclear antigens during the cell proliferation cycle and is therefore a marker of rapid tumor growth. The expression of Ki-67 in breast cancer is significantly related to pathological grade and LN metastasis (15). Furthermore, proliferating cell nuclear antigen (PCNA) is an intranuclear polypeptide synthesized during cell proliferation. During the malignant proliferation of cancer cells, PCNA expression is abnormally high. Studies have shown that high PCNA expression in patients with tumors results in rapid clinical progress and predisposition to LN metastasis (16). In addition, the positive expression of cyclooxygenase-2 (COX-2) in breast cancer tissue is significantly higher than that in other tumor diseases and is correlated with lymphatic and distant metastasis (17).

The aim of this study was to explore a feasible, effective, and non-invasive joint model to assess lymphatic metastasis of patients with breast cancer based on the radiomic features of their T1-weighted image (T1WI)-enhanced scans and post-operative molecular biomarkers. This will contribute to a new quantitative analysis for evaluating the risk of breast cancer LN metastasis before surgery.

## Materials and Methods

### Patients

This study was reviewed and approved by the ethics committee of the Huangshi central hospital. Patients diagnosed with breast cancer *via* pathology in the hospital between June 2015 and June 2019 were strictly screened according to the following inclusion and exclusion criteria, inclusion criteria:1) solid masses in the image of the lesions; 2) maximum diameter of the lesion ≤2 cm; 3) diagnosis of primary breast cancer; 4) MRI shows no axillary lymph node (ALN); 5) SLN before treatment; 6) post-operative tumor tissue identified as breast cancer after pathological examination; 7) twice LN biopsy to confirm the status after operation; 9) non-metastatic primary lesions on pathological examination. Exclusion criteria: 1) breast implants such as silicone; 3) radiotherapy, chemotherapy, drug therapy and surgical treatment before enhanced MRI scan; 4) poor image quality of MRI.

### MRI Scanning

All images were obtained using enhanced MRI scanning (1.5T superconducting MRI; Siemens, Munich, Germany). The patient was required to lie on his back (feet first) and wear noise-reducing headphones for the MRI; the conventional T1WI enhancement sequence in the transverse position was selected, centering on the largest layer of the lesion, and a total of 20 layers were scanned up–and–down. A bolus injection of the contrast agent gadolinium diamine (dose: 0.1 mmol/kg; General Electric Pharmaceuticals, Shanghai, China) was selected, and the injection rate was set at 2 mL/s. After the bolus injection of the contrast agent was completed, 20 mL normal saline was injected at the same rate for flushing. The scanning parameters were set as follows: TR 3.9 ms, TE 1.4 ms, FOV 380 mm × 280 mm, matrix 256 × 256, layer thickness 5 mm, and 20 slices total per volume.

### Molecular Biomarker Analysis

The tissue of breast cancer was collected by needle biopsy. The tissue of breast cancer was fixed by 4% paraformaldehyde and then embedded by paraffin. Immunohistochemistry (IHC) was used to detect the expression of Ki-67, COX-2, PCNA, PR and Her2. for Ki-67, samples with >20% positive nuclei were considered to show high Ki-67 expression, while samples with 20% positive nuclei were considered to show low Ki-67 expression (18, 19); for COX-2, samples with >30% positive cytoplasm were considered to show high COX-2 expression, while samples with <30% positive cytoplasm were considered to show low COX-2 expression; for PCNA, samples with >10% positive nuclei were considered to show high PCNA expression, while samples with <10% positive nuclei were considered to show low PCNA expression; for PR, samples with >1% nuclear staining as PR positive, <1% nuclear staining as PR negative (20); Her2 positive was detected by IHC that the staining score was 3+ (21).

### Image Analysis

The data were processed by two doctors with 10–15 years of diagnostic experience in the following steps. The original MR images were imported into ITK-SNAP (www.itksnap.org), and the breast cancer lesions were processed by the diagnostician according to the single-blind principle. The lesions were then delineated and synthesized in three-dimensions (3D), the whole tumor was segmented, and the 3D region of interest (volume of intervalidation, VOI) was saved. The lymph was not delineated in this study. The image and VOI were then imported into Anaconda Prompt (version 4.2.0) importing the feature package of “pyradiomics” (github.com/Radiomics/pyradiomics), according to the guidelines of the Image Biomarker Standardization Initiative (IBSI). A total of 1,051 features were extracted, including shape parameters, first-order parameters, gray level co-occurrence matrix (GLCM parameters), gray-level run-length matrix (GLRLM parameters), gray level size zone matrix (GLZSM parameters), and gray level dependence matrix (GLDM parameters).

### Statistical Analysis

**Figure 1:**
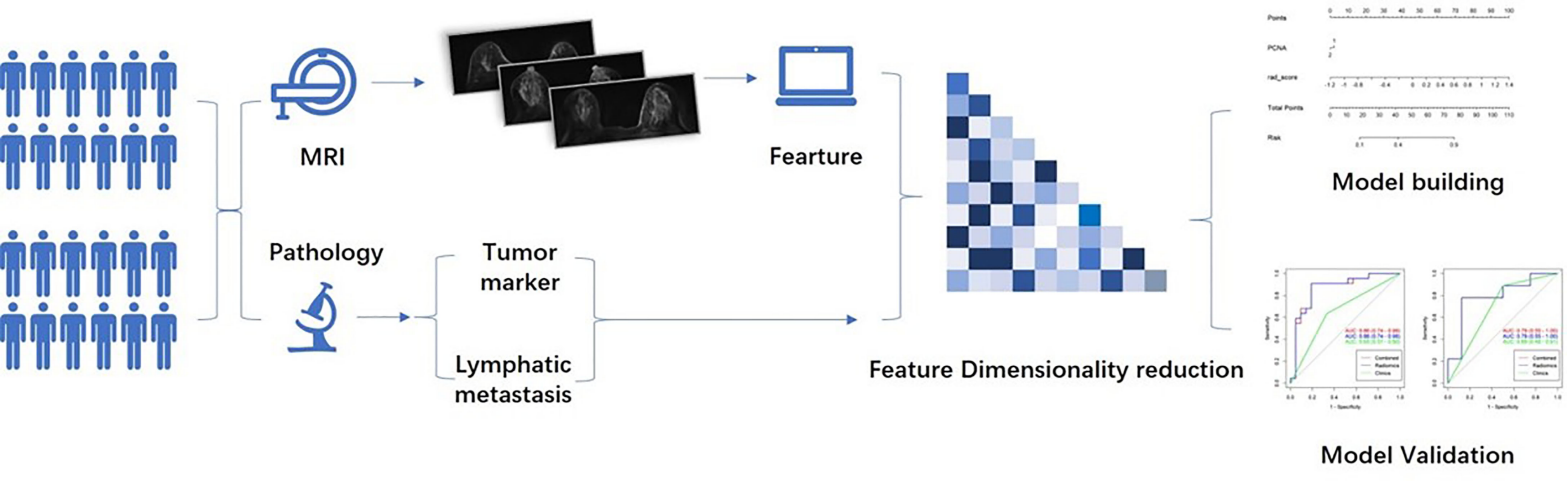
Flowchart depicting model design.

The maximal relevance and minimal redundancy (mRMR) algorithm with the least absolute shrinkage and selection operator (LASSO) method were used for feature dimensionality reduction, whereas the stepwise regression method was used to filter the radiomics features into the multivariate logistic regression analysis to obtain meaningful feature regression coefficients, to perform feature weighting and construct the radiomics label (Rad-score) for TNM staging assessment. Next, the meaningful tumor markers were screened for multiple logistic regression with a Rad-score, using a joint evaluation model, and a nomogram was drawn for the scoring system with the best predictive performance obtained from the above scores. The clinical application value was evaluated by decision curve analysis (DCA). The general process is illustrated in **Figure 1**.

The Kolmogorov-Smirnov test was used to validate normal distribution of the measurement data. Normally distributed data were represented as mean ± standard deviation, and non-normal data were represented by the median. Independent sample *t* validation or Mann-Whitney U validation was used for the measurement data, and *X* ^2^ validation was used for the count data. Differences were considered statistically significant at *P<0.05*. The performance of the scoring system was evaluated based on the area under the ROC curve (AUC). R studio (version 4.1.1) was used for processing and analysis, with specific packages: “xml2,” “tidyverse,” “caret,” “pROC,” “glmnet,” “DMwR,” “rmda,” “ggpubr,” “ModelGood,” “rms,” “mRMRe,” “DescTools,” and “Publish.”

## Results

### Clinical Characteristics Between LN-Positive and -Negative Patients in the Training and Validation Cohorts

A total of 185 patients were included in this study, whereas 100 were included according to the inclusion criteria: 45 patients without LN metastasis and 55 patients with LN metastasis (**Table 1**). Particularly, 30 patients were excluded due to poor image quality, and 55 patients were excluded because of unclear data pertaining to molecular biomarkers. The average age was 52.9 ± 11 years for the patients without LN metastasis and 53.6 ± 10 years for the patients with lymph node metastasis in the training cohort. At same time, the average age was 52.4 ± 12.7 years for the patients without LN metastasis and 51.2 ± 9.8 years for the patients with LN metastasis in the validation cohort. There was no significant difference between the non-LN metastasis and LN metastasis in the training cohorts and validation cohorts (*P=0.769 vs P=0.775*). Among the enrolled patients, 59 were positive for Ki-67 (41negative), 73 were positive for PCNA (27 negative), and 66 were positive for COX-2 (34 negative), and 55 were positive for PR (45 negative), 66 were positive for Her2 (34 negative) Ki-67 (*P=0.050*, **Table 1**) and Her2 (*P=0.004*, **Table 1**) showed significant difference between NLN and LNM in the training cohort, and COX-2 (*P=0.041*, **Table 1**) showed significant difference between NLM and LNM in the validation cohort.

**Table 1:** Clinical information of patients in the training and validation cohort.

	Training cohort		P value	Validation cohort		P value
	NLN	LNM		NLN	LNM	
Patients	n = 32	n = 39		n = 13	n = 16	
Age, years	52.9 ± 11	53.6 ± 10	0.769	52.4 ± 12.7	51.2 ± 9.8	0.775
Histological grade						
I	2	3		1	1	
II	14	12		7	6	
III	16	24		5	9	
Molecular status						
Ki-67			0.050*			0.061
Positive	23	18		11	7	
Negative	9	21		2	9	
PCNA			0.099			0.364
Positive	27	25		11	10	
Negative	5	14		2	6	
COX-2			0.167			0.041*
Positive	24	22		12	8	
Negative	8	17		1	8	
PR			0.144			0.867
Positive	22	19		7	7	
Negative	10	20		6	9	
Her2			0.004*			1.000
Positive	15	32		9	10	
Negative	17	7		4	6	

NLN, non-lymph node metastasis; LNM, lymph node metastasis; *means significant difference.

### The Radiomics Signature of LN-Positive and -Negative Patients in the Training and Validation Cohorts

**Figure 2:**
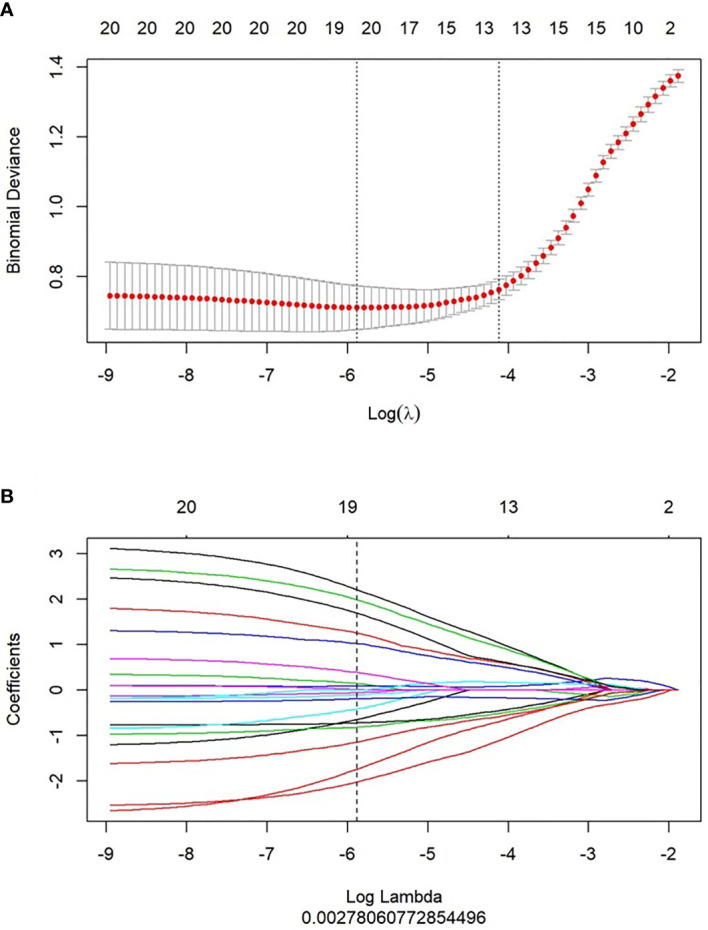
Feature selection and Rad-score building by LASSO. **(A)** 10-fold cross validation was used to predict binomial deviance of the Rad-score building by different lambda values. **(B)** The coefficient profiles of the radiomics features by different lambda values.

**Figure 3:**
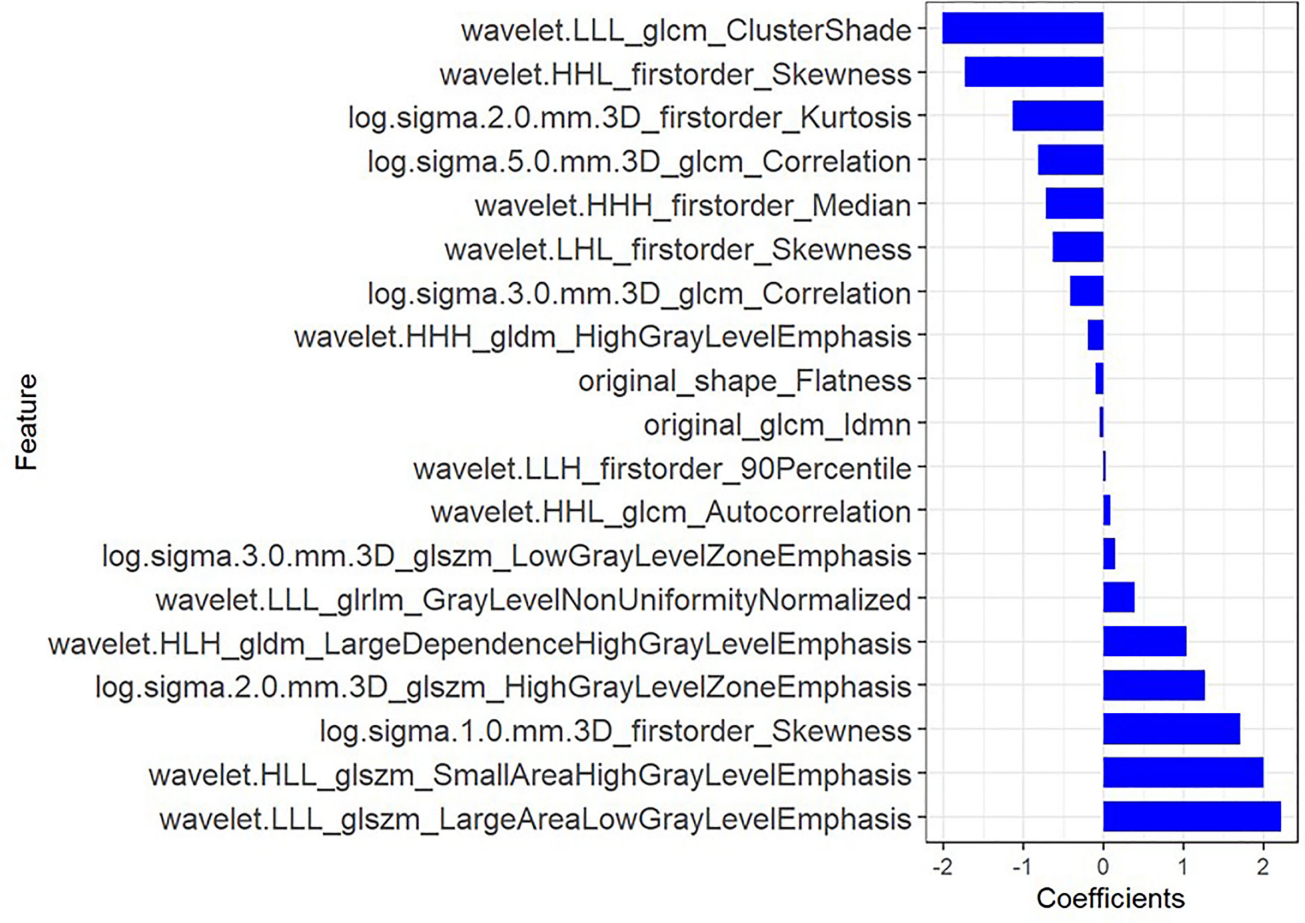
The coefficients of radiomic features to construct the Rad-score.

Using mRMR to remove redundant features and screen out the feature combinations that are significantly different for lymphatic metastasis, a total of 30 features were selected. Next, LASSO was used to reduce the dimensionality of the radiomics features, taking log(λ) as 0.0027 (**Figure 2**), and finally 19 parameters were retained to construct the radiomics. The Rad-score is based on the following formula \(**Figure 3**):


Radscore=−0.179×wavelet.HHH_firstorder_Median−2.016×wavelet.LLL_glcm_ClusterShade+0.15×log.sigma.3.0.mm.3D_glszm_LowGrayLevelZoneEmphasis+0.087×wavelet.HHL_glcm_Autocorrelation−0.414×log.sigma.3.0.mm.3D_glcm_Correlation+0.017×wavelet.LLH_firstorder_90Percentile−0.64×wavelet.LHL_firstorder_Skewness−1.736×wavelet.HHL_firstorder_Skewness−0.817×log.sigma.5.0.mm.3D_glcm_Correlation+1.03×wavelet.HLH_gldm_LargeDependenceHighGrayLevelEmphasis−0.102×original_shape_Flatness−0.056×original_glcm_Idmn+2.212×wavelet.LLL_glszm_LargeAreaLowGrayLevelEmphasis+1.26×log.sigma.2.0.mm.3D_glszm_HighGrayLevelZoneEmphasis+1.992×wavelet.HLL_glszm_SmallAreaHighGrayLevelEmphasis−0.194×wavelet.HHH_gldm_HighGrayLevelEmphasis+0.39×wavelet.LLL_glrlm_GrayLevelNonUniformityNormalized+1.699×log.sigma.1.0.mm.3D_firstorder_Skewness−1.142×log.sigma.2.0.mm.3D_firstorder_Kurtosis+0.508

**Figure 4:**
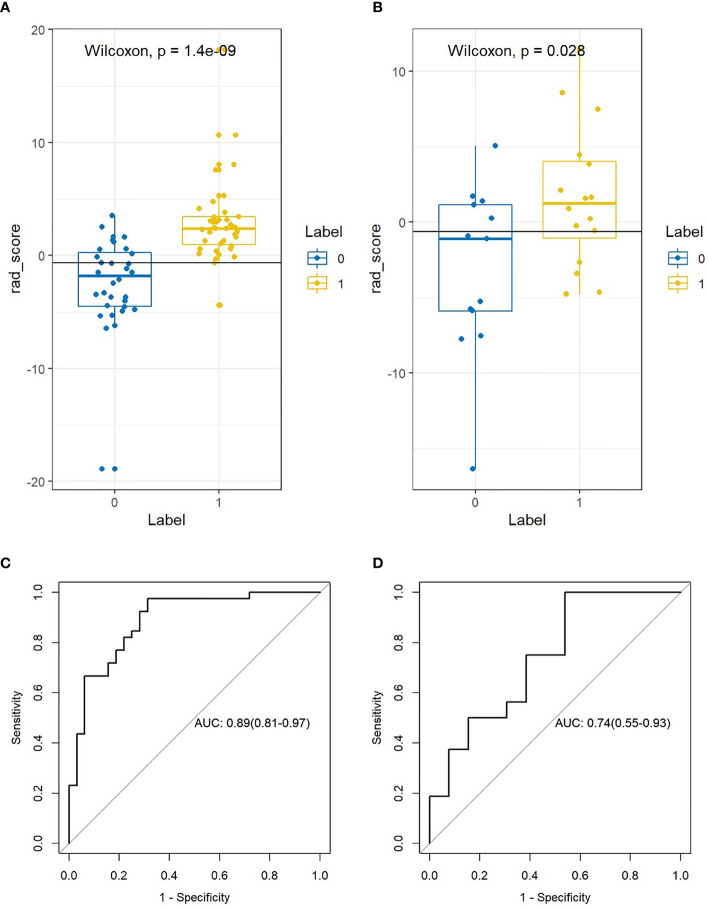
The difference and ROC curves of the Rad-score in the training and validation cohorts. **(A, B)** Mann-Whitney U validation was used to analyze the difference between lymph node (LN)-positive and -negative patients in the training and validation cohorts. **(C, D)** ROC curve of the Rad-score in the training and validation cohort. AUC was used to predict the diagnostic performance between the LN-positive and -negative patients.

After the Rad-score calculation for all patients in both the training and validation cohorts, based on the Wilcoxon validation, there was a significant difference between the non-lymphatic and lymphatic metastasis groups in the training cohort (*P*=0.000, **Figure 4A**) and the validation cohort (*P*=0.028, **Figure 4B**).

### Diagnostic Performance of the Rad-Score in Different LN-Positive and -Negative Patients in the Training and Validation Cohorts

After obtaining the Rad-score of patients in the non-lymphatic and lymphatic metastasis groups, the diagnostic performance of the Rad-score was evaluated based on the ROC. The Rad-score distinguished between non-lymphatic and lymphatic metastasis with an AUC of 0.891 (**Figure 4C**) in the training cohort and 0.744 (**Figure 4D**) in the validation cohort (**Table 2**).

**Table 2:** Diagnostic performance of the Rad-score, clinical data, and nomogram in the training and validation cohorts.

	Cohort	AUC	95% CI		Accuracy	Sensitivity	Specificity	PPV	NPV
			Lower	Upper					
Rad-score	Training	0.891	0.812	0.967	0.845	0.974	0.687	0.792	0.956
	Validation	0.744	0.552	0.931	0.689	0.750	0.615	0.705	0.667
Clinics	Training	0.719	0.602	0.843	0.690	0.820	0.531	0.680	0.708
	Validation	0.588	0.0.379	0.801	0.483	0.625	308	0.526	0.400
Nomogram	Training	0.936	0.882	0.992	0.901	0.974	0.812	0.863	0.962
	Validation	0.793	0.624	0.959	0.603	0.727	1.000	1.000	0.538

95% CI, 95% confidence interval; NPV, negative predictive value; PPV, positive predictive value.

### Diagnostic Performance of the Rad-Score, Clinical Factors, and Nomogram in Different LN-Positive and -Negative Patients in the Training and Validation Cohorts

**Figure 5:**
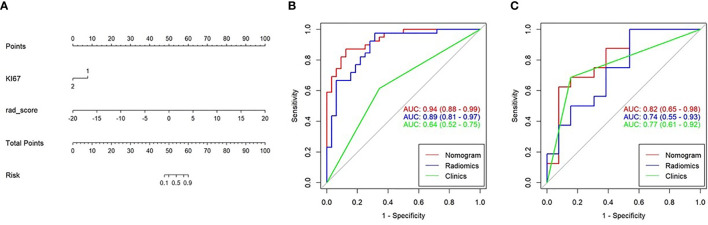
Nomogram combining the clinical data and Rad-score. **(A)** The multiple logistic regression model constructed using the Rad-score and clinical data visualized by the nomogram. **(B, C)** The ROC curves of the Rad-score, clinical data, and nomogram in the training and validation cohorts.

In the training cohort, Ki67 and Her2 showed significant difference between NLM and LNM. Backward step logistic model was used to build clinical model. Ki67 (OR=0.44, 95%CI: 0.15-1.25) and Her2 (OR=4.41, 95%CI: 1.47-13.24) were used to construct the clinical model based minimum AIC. Then the combined model was constructed by molecular biomarker (OR_Ki67 =_ 0.19, 95%CI: 0.03-1.07; OR_Her2 =_ 4.41, 95%CI: 0.97-26.50) and radscore (OR=2.28, 95%CI:1.50-3.46). The combined model was visualized by Nomogram (**Figure 5A**). The evaluation performance of the clinical model for the training and validation cohort were 0.642 and 0.773 (**Figures 5B, C** and **Table 2**). The nomogram showed diagnostic performance for the training and validation cohort were 0.936 (**Figure 5B**) and 0.793 (**Figure 5C** and **Table 2**).

### Evaluation of Lymph Metastasis *via* a Nomogram

**Figure 6:**
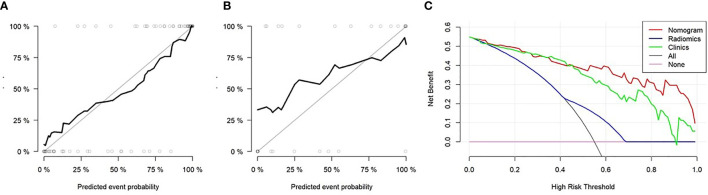
The diagnostic performance of nomogram evaluation. **(A)** Decision curve analysis of the Rad-score, clinical data, and nomogram. The y-axis indicates the clinical benefits while the x-axis indicates the clinical risk to predict lymph node (LN) metastasis. The “All line” indicates a randomized evaluation of the LN metastasis. The pink line indicates no method was used to evaluate the LN metastasis. **(A–C)** Calibration curves of the nomogram in the training and validation cohorts.

The nomogram was used to visualize the combine model. The Rad-score, Ki67 and Her2 score axis were projected vertically to the Points axis, and the total risk for assessing breast cancer lymphatic metastasis was given as the total points of lymphatic metastasis. The greater the risk, the greater is the probability that a patient would present with lymphatic metastasis (**Figures 6A, B**). The DCA analysis for clinical model, Radscore and Nomogram have indicated the threshold under 0.92 that patient would benefit from nomogram (**Figure 6A**). (**Figure 6C**) Delong test have showed a significant difference between nomogram and clinical model in the training cohort (P=0.0001) but not in validation cohort (P=0.111). At the same time, radscore showed a statistically significant difference when compared with clinical model in the training cohort (P=0.027) but also not in validation cohort (P=0.027).

## Discussion

In this study, we performed a non-invasive assessment of lymphatic metastasis in patients with breast cancer based on a multiple logistic regression model that combined the radiomic tag Rad-score extracted from the conventional breast cancer T1WI-enhanced scan with the tumor biomarkers. The results demonstrated that the diagnostic efficiency of Rad-score (training cohort AUC = 0.891; validation cohort AUC = 0.744), was higher than that of the tumor biomarker model (clinical model, training cohort AUC = 0.642; validation cohort AUC = 0.773). However, once Rad-score and molecular biomarker model were combined as nomogram, the diagnostic efficiency was the highest among radiscore and clinical model in the training cohort (AUC=0.936) and validation cohort (AUC=0.793). In the current clinical treatments, lymphatic metastasis is an important prognostic indicator and factor in selecting the appropriate treatment for patients with breast cancer. A study by Wang et al. showed that 44% (95/216) of patients with breast cancer had sentinel lymphatic metastasis (13). However, both the sentinel lymphatic biopsy and axillary LN dissection are invasive diagnostic techniques, whereas MRI can be used as a qualitative imaging assessment method to determine breast cancer lymphatic metastasis (22, 23). Therefore, this study aimed to establish a non-invasive and highly sensitive model for assessment of breast cancer lymphatic metastasis.

First, the texture feature parameters were extracted based on the conventional T1WI enhanced scan, and after removing redundant features, 19 parameters were retained. These parameters can be understood as follows. Usually, Interia_angle45_offset7 reflects the definition of the image and the depth of the texture groove—the higher the groove depth, the higher the image contrast and definition. GLCMEntropy_ AllDirection_offset1_SD reflects the entropy value of the image, which represents the image required for image compression—the higher the entropy value, the higher the confusion of the image. ShortRunEmphasis_angle90_offset1 reflects all voxel points that are not often 1 at a given angle (90°), and Correlation_ AllDirection_offset4_SD represents the similarity of gray levels in adjacent pixels. InverseDifferenceMoment_ AllDirection_ offset4_SD represents local homogeneity, which is proportional to local gray uniformity. The Rad-score value of all patients obtained by the Rad-score calculation formula was as follows: AUC of 0.891 in the training cohort and 0.744 in the validation cohort. Chai et al. (24) showed that based on T1WI, CE2, T2WI, and DWI sequences for radiomics feature extraction, the AUC reached 0.87, which was similar to the results of this study. Simultaneously, the study showed that the vascular permeability parameters *K^trans^*, *K_ep_*, *V_e_*, *V_p_*, etc. could improve the diagnostic performance of the model (accuracy 0.86, AUC 0.91) (24). Currently, according to breast cancer imaging guidelines, dynamic enhanced MRI is used as a clinically recommended protocol for the diagnosis of breast cancer, which suggested that radiomic features should include dynamic enhancement in future studies. Previous studies have showed that the short-term survival didn’t have different between patients who taken ALND or not. The American society of clinical oncology (ASCO) recommended SLNB for patient with early-stage breast cancer to reduce the unnecessary ALND. Previous studied have found that the sensitivity of SLND was 0.855, the sensitivity of Rad-score were 0.974 (training cohort) and 0.750 (validation cohort)

In the second, the gender, age, Ki67, COX-2, PCNA, PR and Her 2 were included in the lymphatic metastasis assessment of breast cancer in this study. The final screening index with statistical significance was Ki67 and Her2. Lymphatic metastasis was assessed based on clinical model combined Ki-67 and Her2, with an AUC of 0.7192 in the training cohort and 0.588 in the validation cohort, which was lower than the diagnostic power of radiomics (AUC = 0.891 and 0.744, respectively). Our results are similar to those of Wang et al. (16), which reported clinical index parameters of 0.707 and 0.657, respectively, to assess breast cancer lymphatic metastasis. However, their clinical indicators were tumor diameter, tumor molecular classification, ER phenotype, etc., which differed from the clinical indicators in this study. These findings suggest that multiple clinical indicators should be included in the assessment of breast cancer LN metastasis (13). In our studies, we included 5 type molecular biomarker try to predict the status of lymph node metastasis. All patients included in our research are ER positive. Our results showed that Ki67 and Her2 were the risk factor of lymph node metastasis. The OR of Ki67 and Her2 were 0.44 and 4.41 which were similar to previous studies (11, 25). In our model, Her2 overexpression would improve the risk of lymph node metastasis. Ki-67 positive also improve the risk of lymph node metastasis. Previous studied showed that Her2 and Ki67 could help in increasing the sensitivity to estimate the probability of lymph node positive (26). Our results showed Ki-67 and Her 2 combined the radscore have improve the sensitivity and specificity in training and validation cohorts. However, these results showed that the nomogram constructed by the Rad-score, integrated with clinical indicators, was more effective in evaluating breast cancer lymphatic metastasis than the clinical indicators alone, although it was the same as the independent Rad-score. This result indicates that the Rad-score has diagnostic power with or without clinical indicators. This means that the diagnostic efficiency of the Rad-score was significantly higher than that of the clinical indicators, and the evaluation of breast cancer lymphatic metastasis could be based on the Rad-score indicators. This result is different from that of the clinical imaging joint model used to improve the diagnostic efficiency (27, 28). The procedure of SLNB consumed time and expensive even through SLNB is the gold standard to diagnosis lymph node metastasis. SLNB would take adverse event through invasive procedure. Nomogram, combined molecular biomarker, also needed invasive biomarker. In our study, Rad-score was constructed to predict LNM before surgery without invasive method.

Potential limitations of this study include the small sample size and the omission of molecular classification of tumors, which might have led to the low efficiency of the clinical indicators for breast cancer. The clinical indicators used in this study were tumor resection and immunohistochemical detection. This suggests that clinical indicators should be expanded to establish a more comprehensive clinical joint model in future studies. In addition, MRI multimodal sequences should be included in the radiomics feature extraction, especially multi-phase dynamic enhancement.

In conclusion, a large number of radiomic parameters were extracted based on conventional T1WI MRI enhancement in this study, and a radiomics model was constructed to evaluate breast cancer lymphatic metastasis. The results demonstrated that the radiomic model had high diagnostic feasibility and efficacy, and the MRI radiomics model might be helpful in evaluating the clinical prognosis of patients with breast cancer.

## Data Availability Statement

The raw data supporting the conclusions of this article will be made available by the authors, without undue reservation.

## Ethics Statement

The studies involving human participants were reviewed and approved by ethics committee of the Huangshi Central Hospital. The patients/participants provided their written informed consent to participate in this study.

## Author Contributions

Guarantor of integrity of entire study, XQ and CC. Study concepts/study design or data acquisition of data analysis/interpretation, XQ. Manuscript drafting or manuscript revision for important intellectual content, all authors. Manuscript final version approval, all authors. All authors agree to ensure that any questions related to the work are appropriately resolved. Literature research, XQ, YF, and CC. Clinical studies, XQ and YY. Statistical analysis, XQ and ZW. Manuscript editing, CC. All authors have read and approved the final manuscript.

## Funding

Hubei Health Committee General Program and Anti-schistosomiasis Fund during 2019-2020 [Grant No. WJ2019M043].

## Conflict of Interest

The authors declare that the research was conducted in the absence of any commercial or financial relationships that could be construed as a potential conflict of interest.

## Publisher’s Note

All claims expressed in this article are solely those of the authors and do not necessarily represent those of their affiliated organizations, or those of the publisher, the editors and the reviewers. Any product that may be evaluated in this article, or claim that may be made by its manufacturer, is not guaranteed or endorsed by the publisher.

## Abbreviations

3D, three-dimensional; AUC, under the ROC curve; COX-2, cyclooxygenase-2; DCA, decision curve analysis; GLCM, gray level co-occurrence matrix; GLDM, gray level dependence matrix; GLRLM, gray-level run-length matrix; GLZSM, gray level size zone matrix; IBSI, Image Biomarker Standardization Initiative; LASSO, least absolute shrinkage and selection operator; LN, lymph node; MRI, magnetic resonance imaging; mRMR, maximal relevance and minimal redundancy; PCNA, proliferating cell nuclear antigen; T1WI, T1-weighted image; VOI, volume of intervalidation.
